# Missing in Action: Sex and Gender in Substance Use Research

**DOI:** 10.3390/ijerph17072352

**Published:** 2020-03-31

**Authors:** Lorraine Greaves

**Affiliations:** 1 Centre of Excellence for Women’s Health, Vancouver, BC V6H 3N1, Canada; lgreaves@cw.bc.ca; 2 School of Population and Public Health, Faculty of Medicine, University of British Columbia, 2206 East Mall, Vancouver, BC V6T 1Z3, Canada

**Keywords:** sex factors, gender, substance abuse, drinking, alcohol, nicotine, research

## Abstract

Substance use and misuse is a significant global health issue that requires a sex- and gender-based analysis. Substance use patterns and trends are gendered: that is, women and men, girls and boys, and gender-diverse people often exhibit different rates of use of substances, reasons for use, modes of administration, and effects of use. Sex-specific effects and responses to substances are also important, with various substances affecting females and males differentially. Nevertheless, much research and practice in responding to substance use and misuse remains gender blind, ignoring the impacts of sex and gender on this important health issue. This special issue identifies how various aspects of sex and gender matter in substance use, illustrates the application of sex- and gender-based analyses to a range of substances, populations and settings, and assists in progressing sex and gender science in relation to substance use.

## 1. Introduction

Substance use is a significant global health issue, with over 271 million people using illicit drugs each year [1], 2.3 billion using alcohol [2], and 1.4 billion using tobacco [3]. The consumption of substances, both licit and illicit, whether misused or not, can affect the mental and physical health and economic status of individuals, families, communities and bystanders, contribute to overall mortality and morbidity, and pose costs to health care and criminal justice systems. Substances can also be used to experience pleasure, respond to trauma, decrease pain, enhance social and cultural bonds, cope with stress, and adapt to problems in living. Hence, responding to substance use is complex, recognizing both health and social costs as well as patterns and motivations among those who use substances. There is a wide range of substances in use, including alcohol, nicotine, cannabis, opiates, and methamphetamines, as well as misuse of prescription drugs. 

Sex and gender both affect substance use in critically important ways. Sex refers to biologically based factors characteristic of female and male bodies, such as hormones, genetics, anatomy, physiology, and organ function. These factors affect how particular bodies respond to the ingestion of substances such as alcohol, cannabis, opioids or nicotine, including the quantity required to create intoxication or effect, create harm or dependence, or cause damage to the body or brain. These factors also affect the range and frequency of resulting health conditions or diseases, such as cancer, heart disease, respiratory illnesses, or psychoses. Sex-specific factors also affect responses to therapeutics and other treatments. These aspects of understanding sex in its entirety are not new. The Institute of Medicine outlined the importance of sex in 2001 for all health researchers and made the fundamental point that “every cell has a sex” [4].

Gender refers to a host of socially and culturally determined factors that affect how men, women, boys, girls and gender-diverse people experience roles, relations, opportunities, customs and expectations. These factors affect individual and social understandings of femininity, masculinity, transgender identities and gender diversity, and are closely tied to cultural contexts. Gender roles and relations affect prevalence and consumption trends, access to substances, initiation into use, responses to advertising, marketing and promotion of legal substances, and how substance use functions in responding to gendered experiences of trauma, caregiving, poverty, social bonding, inequality or marginalization. Gender also affects responses to treatments, policies and health promotion or harm reduction messages. By its nature, gender reflects temporal and cultural interpretations of being men, women or gender diverse, and requires social science theorizing and conceptual development to hone the understanding, use and measurement of gender in its varied contexts.

Taken together, it is easy to see that both sex and gender comprise multiple elements that affect substance use and responses to substance use, such as policy, prevention, or practice. It is important to note that sex and gender also *interact* with each other to affect substance use, and its effects. Sex and gender also intersect with a range of other factors and characteristics such as sexual orientation, income, geography, age, ability, Indigenous status, religiosity, and culture, among others. The measurement challenges posed by these complexities have not been resolved, but these hurdles should not deter substance use researchers from pursuing the impact of sex, gender and sex-gender interactions in our work.

To assist with this process, I have suggested a breakdown of the elements comprising sex and gender for conceptual clarity in order to guide research, practice and policy making in health and in the substance use field. For diagrams, specific examples and to see its application in cannabis, see Greaves and Hemsing [5], in this issue. Sex, for example, includes the impact of hormones, genetics, physiology and anatomy, among many other biologically based aspects of human bodies. Identifying the element of interest in a study assists in sorting out research questions and agendas, clarifying use of terms and language, refining measurement and honing treatments and therapeutics. 

It has been important, for example, to note that female and male responses to alcohol are different, and that females require less alcohol to become inebriated or to sustain damage. This is because female bodies typically have more fat, water and fewer enzymes for breaking down alcohol [6]. Hence, in 2012, Canada adopted sex-specific Low-Risk Alcohol Drinking Guidelines that encourage girls and women to drink less per day, week and sitting than men [7]. This not only had a direct impact on health promotion messaging, but also on clinical assessments of health behavior and on calculations of the rates of risky drinking in Canada. In fact, reducing the level of safe drinking for women immediately created a much wider risky drinking problem than had previously been considered [8]. Clearly, precision about such sex-specific effects also needs to be incorporated into all research designs, measures and analyses, to better inform programming and policy development.

Gender also breaks down into a wide range of aspects, but of primary interest are gender relations, gender roles and gender norms as they affect experiential opportunities in any given social context. The experiences of females and males are impacted by gendered attitudes and the assignment of gender roles and norms resulting in stereotypes deemed congruent with that particular sex. This has global ramifications that include more income inequality for women, less decision-making power in relationships, families and state apparatus, more unpaid work, and more subjugation via intimate partner violence, female genital mutilation (FGM), forced marriage and related concerns. The experiences of men include more familial and political authority, paid work and income, along with more subjection to criminal violence, injury and war experiences. Also of interest is the impact of gender identity. One’s adherence and identification with dominant interpretations of masculinity, femininity, or gender-diverse identities, such as transgender, non-binary or other culturally specific gender-diverse groups, has an effect on how one performs gender, and is directly related to the patterns and prevalence of substance use. For example, gender minority status is often aligned with higher prevalence of substance use. 

Sexual orientation is not to be confused with gender identity. Sexual orientation refers to the romantic and sexual preferences of individuals, and all gender identities can experience a range of sexual orientations including gay, lesbian, bisexual and pansexual. Of interest to the substance use field, though, is that sexual minority status is also a clear risk factor for higher prevalence of substance use. 

Ultimately, applying sex and gender to substance use will facilitate the development of tailored treatment, personalized medicine, targeted policies and more precise health promotion and harm reduction. While the past twenty years has seen a rise in interest and application of sex and gender science in health, the substance use field has been slow to insist on the inclusion of these concepts. This special issue [5,9,10,11,12,13,14,15,16,17,18,19,20,21,22,23] is aimed at providing a remedy for this lack, and more importantly, to provide examples of the impact of sex and gender in a range of substances, cultures, populations and contexts.

These articles address concepts [5], different substances such as alcohol [12,19,23], cannabis [5,17,18], illegal drugs [13], nicotine [11,12], opioids [14], and multi drug use [10], populations such as gay and bisexual men [13], fathers [20], mothers [15], pregnant women [9,23], patients [9,17] and adolescents [14]. They address interactions with interpersonal violence [22], treatment responses [21], abortion policy [23], other harms [12] in a range of settings in countries including Spain, Scotland, Turkey, Canada, China, Taiwan, and the USA. All of the articles address some aspect of sex or gender, interactions between sex and gender, or gender-transformative responses [19] and all contextualize the discussion in a range of cultures and settings. Taken together, they provide a wide range of illustrations of how sex and gender affect both substance use and our responses to it, as well as how research can better address these concepts to derive important and meaningful evidence on which to build treatment and policy.

## 2. Issues with Language and Conceptual Clarity

The research literature has consistently demonstrated that sex and gender are often conflated both linguistically and conceptually, avoided altogether in research design or policy, or measured, but not reported upon or analyzed. In all cases, important understandings are missed or lost that could assist consumers, researchers, practitioners and policy makers in responding to substance use in more useful, accurate and ethical ways. This theoretical and conceptual “muddle” [24,25] is evident in the substance use literature.

For example, in preparing for the systematic review described in detail in Hemsing and Greaves [18] in this issue, aimed at identifying relevant evidence on four drugs (alcohol, cannabis, nicotine and opioids) that reflected on sex and/or gender, a team of researchers at the Centre of Excellence for Women’s Health encountered several issues. We had intended to do a quality appraisal of each selected study and to use Morgan et al. Feminist Appraisal Tool to assist with the appraising of gender, as an additional filter prior to drawing conclusions [26]. However, as described, the response to our search was over 20,000 articles. Once we sifted through these, we found that the muddy conceptual use of sex and/or gender prevented us from confidently carrying out our intended overarching systematic review. Instead, based on this material, we developed several scoping reviews [5,16,18,19] and one small systematic review [17] because terms were either misused or imprecise, or in some cases conflated or used in contradictory ways in a single article. Our intention for systematically surveying the current evidence on sex, gender and substance use was undermined because precise engagement with sex and gender in research was, in essence, missing in action. Some examples of the issues we found include:

1. Using gender to refer to sex or using sex to refer to gender or using both interchangeably.
2. Collecting sex-specific or gender-specific data in the research design, but not reporting it.
3. Collecting sex- and/or gender-related data and reporting but not interpreting or analyzing them.
4. Collecting, reporting and analyzing sex/gender-specific data, but not discussing the impact on practice or policy.

These problems were so ubiquitous that the objective of appropriate analysis was undermined. But these issues are not unique to the literature we searched on substance use. Indeed, these issues are present in much of the extant health literature. While sex and gender science is growing, and more training and resources are available to guide researchers, peer reviewers and students [27], policy makers [28], and practitioners [29,30], there is considerable room for improvement and precision.

## 3. Implications for Substance Use Research, Practice and Policy 

Clearly, it is time to raise the bar on integrating sex and gender more precisely and consistently in substance use research. The articles in this special issue illustrate investigations and analyses on various aspects of sex and gender and substance use in a variety of populations and settings. All of them address sex and/or gender in some way. In addition, one addresses a sexual minority. While not all of the authors identify exactly which aspect of sex or gender is being addressed, readers can, after reviewing the definitions and diagrams [5], and the detailed gender-based analysis addressing cannabis use [18], begin to place the various studies in more precise groups. 

Hubberstey et al. [15] consider the gendered social determinants of substance use among disadvantaged women in order to analyze the nature of help required, focusing on the gendered roles, norms and relations affecting women with children who use substances. Andrews et al. [21] offer an elegant description of the links between early intimate partner violence(IPV) and hypothalamic-pituitary-adrenal axis (HPA) dysfunction, relational deficits and pathways to substance use among women with substance use issues. Essentially, this is a study of sex and gender interactions, focused on a high-risk population of women and children. Motz et al. [22] build on this by demonstrating how the *Breaking the Cycle* program in Toronto, Canada, is constructed to repair these effects of early IPV on women who use substances and their children. 

O’Donnell et al. [20] consider the gendered aspects of creating smoke-free homes for children through a scoping review on the nascent area of engaging fathers in such activity, and highlights the barriers and facilitators that fathers might face. Focusing on fathers and caregiving is a step toward more gender-transformative approaches to smoke-free home initiatives that have traditionally focused on mothers. Wolfson et al. [19] directly assess the gender-transformative impact of brief interventions on alcohol, via a cogent sex and gender analysis of existing literature in that area. Similarly Stinson et al. [16] address the gendered use of technology to respond to substance use treatment and interventions. 

Minian et al. [10] examine a treatment intervention assessing the impact of an online treatment for alcohol use in smokers, and then analyze these results by sex and gender to check for differential outcomes. Similarly, Brabete et al. [17] examine the paucity of evidence on sex-related factors affecting treatment for Cannabis Use Disorder, highlighting the need for research attention on this issue. Font-Mayolas et al. [11] perform a sex disaggregation of descriptive prevalence data on various routes of nicotine administration comparing Turkish and Spanish university students. Their preliminary descriptions and survey questions regarding reasons for use establish basic descriptions of behavior by sex category and invite a two-country comparison. Li et al., from Taiwan [13], address illicit substance use among sexual minority men and the interaction with dynamics such as homophobic bullying and cyberviolence. 

The journey towards more precise usage of sex and gender concepts and terminology is ongoing. However, it is not a frill or an option when designing, doing and reporting on research that is intended to influence policy and practice in the field. Knowing the sex-specific tolerance limits and effects of various substances such as alcohol or cannabis on male and female bodies, is essential to designing accurate and relevant prevention, harm reduction, health promotion, and treatment. Understanding the differential health impacts and diseases that result for males and females from using substances is critical for improving diagnoses, medical care and for refining treatment protocols and programs.

Understanding that gender roles, norms and identities have a direct impact on how and why substances are used, how often, how they are accessed, and what their consumption might mean to users is critically important to designing treatment, policy or prevention. Gender also affects all of our responses to the marketing and promotion of legal substances, and what meanings we associate with substances such as cigarettes, e-cigarettes, alcohol and, in some jurisdictions, cannabis. Further, our gender identity affects how health care practitioners respond to us, diagnose and treat us, often based on stereotypes about masculinity and femininity. Knowing that gender minorities experience above average rates of substance use alerts us to the impact of both gendered and transphobic stigma and discrimination. 

Taken together, sex and gender contribute to explaining the generally lower prevalence of substance use among women, and the higher rates and amounts of use by men. They may also explain the different subjective effects reported by men and women, and boys and girls, of substances such as cannabis or nicotine. Knowing that men experience higher rates of substance use in general alerts us to the impacts of masculinities.

Knowing that those in sexual minorities are consistently at higher risk for use alerts us to the overarching effects of minority stress, homophobia and discrimination. Knowing that these patterns are also gendered, in that bisexual girls and women are the group at highest risk for substance use is a clue as to how important it is to modify our substance use response systems to improve both gender and health equity.

In short, the field of sex and gender science has a lot to offer to the field of substance use. This special issue is an attempt to fill the gaps in the field, where all too often researchers ignore these concepts and factors in designing, doing and reporting on research. These gaps have a direct and negative impact on the health of individuals and groups, and on the ability to be effective practitioners in substance use treatment and prevention.

## 4. Conclusions

Integrating sex and gender analyses into research design not only improves science [31], and increases reproducibility, efficiency and accuracy [32], but also advances social justice and gender equity [26,33]. There are numerous issues of design and measurement that are yet to be resolved [34] and there are considerable challenges in designing methods and measures to address sex and gender and interactions with health determinants [35,36]. Nonetheless, it is heartening to see more research funders in several countries ask for sex and gender concepts to be included in research proposals and offer training for researchers and peer reviewers. It is important that some governments insist on sex- and gender-based analyses of policy and programming (see, for example, Sweden [37], and New Zealand [38]), and offer resources and examples to educate. It is critically important that some journals have adopted sex and gender equity in research (SAGER) [39] guidelines to uphold basic requirements for reporting sex and gender in the research articles that they publish. And, it is critical that implementation and knowledge transfer of evidence includes sex and gender [40]. All of these measures blend together to advance sex and gender science, and to raise the bar on the quality of health research. It is my hope that this special issue will do the same for the field of substance use. 
